# Hallux Valgus Repair with Chevron Osteotomy Significantly Narrows Forefoot Width

**DOI:** 10.3390/jcm12072607

**Published:** 2023-03-30

**Authors:** Raphael Lotan, Benzion Shlomov, Amit Dotan, Alex Bermant, Oded Hershkovich

**Affiliations:** 1 Department of Orthopedic Surgery, Wolfson Medical Center, Holon 5822012, Israel; 2 Sackler Faculty of Medicine, Tel Aviv University, Tel Aviv 6997801, Israel

**Keywords:** hallux valgus (HV), chevron osteotomy, forefoot width, shoe size

## Abstract

Background: Hallux valgus (HV) is a common adult foot deformity. There is uncertainty concerning the effect of HV surgery on foot width. We examined the effect of chevron first metatarsal osteotomy on forefoot width using calibrated pre and postoperative standing radiographs. Methods: A retrospective cohort of 50 patients underwent chevron osteotomy HV surgery. All had HVA > 30°, IMA > 11°, DMMA > 3°, >6-month follow-up, and calibrated pre and postoperative standing foot radiographs. Bony width (BW) and soft tissue width (STW) were used to measure the surgery’s effect on foot width. Measurements were made preoperatively and 3–6 months following surgery. Results: The study group included 42 women with an average age of 63.4 (±8.3) and a mean BMI of 28.7 (±4.9). Preoperative HVA and IMA were 31.7° (±6.8°) and 13.4° (±2.8°), respectively. Following surgery, HVA and IMA improved significantly, by 15.6° (±5.7°) and 8.7° (±2.3°), respectively. The preoperative average BW was 9.4 cm (±0.6), and the STW was 10.6 cm (±0.7). Following surgery, significant changes in BW and STW were measured, with a mean narrowing of 1.2 cm (±0.4) in BW (*p* < 0.001) and 0.95 cm (±0.5) in STW (*p* < 0.001). Paradoxically, an increase in age led to a lower correction of the IMA (*p* = 0.04, r = 0.57), but higher BW and STW reductions (*p* = 0.01, r = 0.35 and *p* = 0.008, r = 0.37, respectively). Conclusions: This study reinforced chevron osteotomy as a valid treatment option that significantly narrows forefoot width; it is thus expected to improve cosmetic outcomes, shoe selection options, and quality of life. This study also found that older age correlates with better forefoot narrowing following hallux valgus repair, possibly due to stiffer soft tissues.

## 1. Introduction

Hallux valgus (HV) is one of the most common adult foot deformities, and affects about a third of the population [1]; its incidence increases with age and mainly occurs in women. The deformity is often asymptomatic, but common complaints are pain and swelling in the bunion area that increases with walking, limitation in fastening narrow shoes, and cosmetic disorder. In light of this, the incidence of deformity correction surgeries is increasing in the population [1].

There are several etiologies for the formation of HV, considered by most as multifactorial, with a genetic tendency in approximately 70% of cases [2]. Hypermobility of the first ray is one of the predisposing factors for the development of hallux valgus (HV), especially if extrinsic risk factors exist [3,4]. Known external risk factors include an injury of the plantar ligament, a tendon–muscle imbalance, and the use of high-heeled shoes [5]; therefore, most patients who seek treatment are women.

The intermetatarsal angle (IMA), hallux valgus angle (HVA), and distal metatarsal articular angle (DMAA) are three crucial measurements used to assess the severity of, and surgical planning for, hallux valgus deformity. The IMA is the angle formed between the longitudinal axes of the first and second metatarsals, indicating the degree of metatarsal splay. The HVA, measured between the longitudinal axes of the first metatarsal and proximal phalanx of the hallux, represents the degree of deviation of the great toe from its normal anatomical position. Lastly, the DMAA is the angle formed between the articular surface of the distal first metatarsal and the longitudinal axis of the same bone. This angle reflects the orientation of the distal metatarsal articular surface and is crucial in determining the need for distal metatarsal osteotomy during surgical correction. The American Foot and Ankle Society grades HV as moderate to severe with an HV angle (HVA) above 30 degrees, intermetatarsal angle (IMA) more than 11 degrees, and distal metatarsal articular angle (DMAA) above 15 degrees [6].

A review by Easley and Trnka et al. [7,8] previously described 100 different surgeries to repair HV deformities, implying that there is no perfect procedure or a single surgery that provides an excellent solution to all types and degrees of deformation. A logical decision-making algorithm was developed to assess and treat patients with HV. The deformity is divided into three groups based on X-ray images: congruent joints, incongruent joints (subluxated), and arthritic joints [9,10].

The chevron osteotomy is one of the most common distal osteotomies used, and was first described by Leventen and Corless [11,12]. It is a “V” shaped osteotomy at the neck of the first metatarsal, which allows lateral displacement of the metatarsal head. Its advantage is a stable osteotomy with a large osseous contact area between edges that increases the chances of fusion without shortening or over-alignment of the first metatarsus, thus diminishing one possible complication, transfer metatarsalgia. The procedure is associated with excellent results in mild to moderate HV deformity [13].

Evaluation of HV surgical results usually includes a physical examination, radiographic IMA and HVA differences pre- and post-surgery, and questionnaires, such as the American Orthopedic Foot and Ankle Society (AOFAS) MetaTarsoPhalangeal-InterPhalangeal Score [14]. HVA and IMA measurements by the same or different surveyors can vary by more than 5 degrees [15]. Thordarson et al. [16] reported a mismatch between radiographic and functional results; therefore, radiographic evaluation was insufficient, requiring physical examination and patient satisfaction assessment.

A significant preoperative concern is the foot’s appearance after surgery and the ability to wear narrow shoes without limitation. Mann et al. [17] found that 60% of patients were mainly concerned about the cosmetic appearance of the foot following surgery. The patient’s perception of surgical success is directly related to the postoperative foot’s shape [18,19], with most (41–62%) patients regaining the ability to wear high heels or narrow shoes following HVR [2,17]. The cosmetic result and unlimited shoe wear are essential factors in patient satisfaction with surgery beyond mechanical/radiographic repair.

There is uncertainty concerning the effect of HV surgery on foot width. Several studies examined the impact of HV surgery on foot width, but most did not utilize calibrated radiographs, leading to non-quantitative measurements. Conti et al. [16] measured calibrated radiographs following Lapidus proximal first metatarsal osteotomy and found a narrowed foot width. Similar studies examining the effect of diaphyseal Scarf osteotomy found that this surgery did not result in a significant narrowing of foot width, and in some cases even caused a widening of foot width [17].

This study aimed to examine the effect of chevron first metatarsal osteotomy on forefoot width using calibrated pre and postoperative standing radiographs.

## 2. Methods

We conducted a retrospective study, including patients who underwent HV surgery due to a medium to severe hallux valgus deformity between 2019 and 2020 at our orthopedic surgery department. Inclusion criteria included: above 18 years old, HVA > 30°, IMA > 11°, DMMA > 3°, surgery performed was a first metatarsal neck chevron osteotomy with or without an Akin osteotomy, more than six months follow-up, and calibrated pre and postoperative standing foot radiographs. In this procedure, a “V” shaped osteotomy of the distal metatarsal is made, which allows the first MT head to be transferred laterally, correcting the abnormal figure.

Exclusion criteria included rheumatic diseases (gout, rheumatoid arthritis, and psoriatic arthritis), pes planovalgus, previous foot fractures, revision surgery, active infection, and malignancy involving the foot.

**Figure 1:**
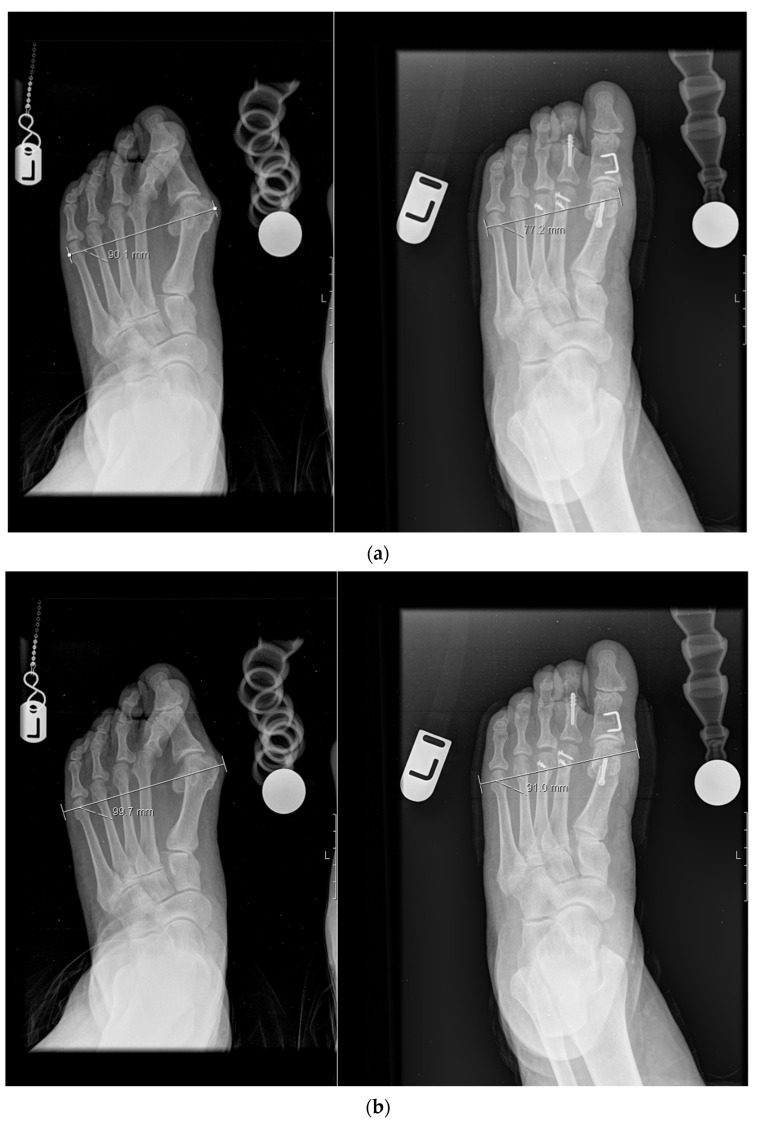
Pre and postoperative measurements of (**a**) BW and (**b**) STW on standing calibrated X-rays.

Patients’ demographic data (age, gender, BMI, and background diseases) were collected. Bony width (BW) was used to measure the surgery’s effect on foot width and maximal distance between the medial cortex of the first metatarsal head and the lateral cortex of the fifth metatarsal head (Figure 1a). Soft tissue width (STW) was measured using the maximal distance between the medial soft tissue border at the height of the first metatarsal head and the lateral soft tissue border of the fifth metatarsal head (Figure 1b). Measurements were made preoperatively and 3–6 months following surgery (Figure 1). All X-rays were upright calibrated using a calibration ball set on the floor. This measurement method has limitations, as the intermetatarsal distance is inclined to the foot axis and may vary due to first metatarsal lengthening or lateralization. This method was used in previous studies and was chosen to facilitate comparing results.

## 3. Statistical Analysis

Statistical analysis was performed using SPSS software version 25 (IBM SPSS Statistics for Windows, Version 25.0. Armonk, NY, USA: IBM Corp). Data were evaluated for normal distribution using the Kolmogorov–Smirnov test. Descriptive statistics were calculated using averages and standard deviations for the quantitative research variables and incidence tables for the categorical research variables. Univariate analysis was performed using the Wilcoxon signed ranks test. The Mann–Whitney test was performed for independent samples. The Spearman correlation test was calculated for quantitative research variables. Finally, multivariate linear regressions were performed according to the principle of the ordinary least-squares regression. Findings were considered significant if their *p*-values were less than 5%.

## 4. Results

During 2019–2020, eighty-three HVD surgeries were performed at our center. We included fifty cases in the study according to the inclusion and exclusion criteria. The 33 excluded patients included those who had surgeries by various methods (Scarf osteotomy, MICA, metatarsophalangeal fusion, or single bunionectomy), patients lost for follow-up, or who lacked pre and postoperative surgical radiographs.

Thirty-three patients underwent surgery on the right foot compared to seventeen on the left foot. The study group included, as expected, mainly women (42 vs. 8); the average age was 63.4 (±8.3). Most patients were overweight, with a mean BMI of 28.7 (±4.9) (Table 1). The average time from surgery to weight-bearing foot X-ray was 4.8 ± 1.1 months. The Shapiro–Wilk test of normality proved that age (*p* = 0.09), BMI (*p* = 0.27), HVA (*p* = 0.89), IMA (*p* = 0.53), BW (*p* = 0.13), and STW (0.36) had normal distributions.

Average preoperative HVA and IMA measurements were 31.7 (±6.8) and 13.4 (±2.8) degrees, respectively, corresponding to a medium to severe hallux valgus deformity. Following surgery, HVA and IMA measurements were significantly improved, by 15.6 degrees (±5.7) and 8.7 degrees (±2.3), respectively (*p* < 0.001) (Table 2).

Preoperative average bone width (BW) was 9.4 cm (±0.6), and soft tissue width (STW) was 10.6 cm (±0.7). Following surgery, significant changes in BW and STW were measured, with a mean narrowing of 1.2 cm (±0.4) in BW (*p* < 0.001) and 0.95 cm (±0.5) in STW (*p* < 0.001) (Table 2). In one case, STW did not change after surgery. Gender was not statistically significant in HV measurements before or after surgery (Table 3).

After studying the effect of BMI, HVA, IMA, gender, and side of surgery on the extent of postoperative changes in BW and STW, the only parameter found to be statistically significant in a multivariate analysis was the patient’s age, which was a positive predictor of BW and STW narrowing (*p* = 0.01) (Table 4 and Table 5). Paradoxically, an increase in age led to a lower correction of the IMA (*p* = 0.04, r = 0.57), but a higher BW and STW reduction (*p* = 0.01, r = 0.35 and *p* = 0.008, r = 0.37, respectively). 

## 5. Discussion

Numerous publications deal with hallux valgus deformity repair options and measure the average correction achieved by various surgeries [7,8]. Previous studies have demonstrated that foot appearance and the comfort of choosing footwear impact patients’ satisfaction with surgery. Saro et al. [18] found that deformity correction did not correlate with quality of life, whereas footwear options were positively affected. Schneider et al. [19] suggested that a decrease in pain and improvement in the choice of footwear were significant preoperative patient expectations that needed addressing. McRitchie et al. [20] reported that 65% of their patients who sought treatment due to HV deformity wore after-surgery shoes that were the original, or less than the original, width of their foot. Tai et al. [21] showed that postoperative foot shape was among the top ten priorities for surgical outcomes in women. These studies support the understanding that postoperative foot width significantly affects footwear options, especially for women who prefer narrow-toe compartment shoes.

Although a simple bunionectomy surgery reduces forefoot width independently, a previous study [22] showed that hallux valgus surgery increases forefoot width. This result differs from the expected effect of chevron osteotomy for hallux valgus, leading to further radiographic investigation of surgical results.

In this study, HV deformity repair was performed using the chevron-shaped osteotomy. Postoperative HVA and IMA measurements showed an impressive deformity correction, with an average of 16.1 degrees and 4.7 degrees, respectively, or 50.9% and 35.1%, respectively (*p* < 0.001). These data illustrate the efficacy of chevron osteotomy in hallux valgus deformity correction, as previously described [7,8,17,19]. Beyond mechanical correction, this method achieved significant forefoot narrowing, with an average osseous narrowing of 1.2 cm (±0.4), and 0.95 cm (±0.5) in soft tissue width (*p* < 0.001). The STW did not change in one case, despite a 0.4 cm BW narrowing and HVA and IMA correction. We suspect that this minor preoperative deformity did not mandate a significant first metatarsal head displacement, thus leading to insignificant narrowing of the forefoot.

Shoes are manufactured with different toe box sizes, ranging from narrow (AA), medium (M or B), wide (D), and extra-wide (EE) toe boxes. The average width difference between each category is 0.95 cm in women’s footwear and 0.48 cm in men’s footwear [23]. In our study, HV repair using the chevron osteotomy reduced forefoot soft tissue width by an average of 0.95 cm (±0.5); thus, reductions of one shoe size for women and two shoe sizes for men were achieved. As a positive correlation was found between shoe size and HV repair results, such a significant postsurgical foot narrowing is expected to improve overall satisfaction.

We found no statistical significance regarding the effect of BMI, the severity of preoperative deformity (HVA and IMA), or patient gender on the degree of forefoot narrowing following chevron osteotomy. Interestingly, we found a statistically significant correlation between patient age and surgical outcome. However, the degree of IMA reduction decreased with age, and the magnitude of bone and soft tissue narrowing increased. This relationship can be attributed to age-related changes in soft tissue quality; young patients have flexible soft tissues compared to older patients, thus leading to greater splaying of the foot despite better osseous correction. Load-bearing foot mobility differences by age were previously described in 3D studies [24,25]; older feet have less mobility, as was found in our study. On the other hand, older patients with less flexible soft tissues maintained better forefoot narrowing with lesser osseous correction. These findings correlate with other publications regarding hypermobility as an influencing factor in the development and outcome of correction for HV deformity [3,26].

The limitations of our study include its retrospective design, a relatively small sample size, the lack of a control group to compare other repair methods, and the limitation of the radiographic measurement techniques used. In addition, the study focused on radiographic indices without correlation to patients’ pain, satisfaction, or quality of life.

## 6. Conclusions

This study reinforced chevron osteotomy as a valid treatment option that significantly narrows forefoot radiographic width; thus, it may improve cosmetic outcomes, shoe selection options, and quality of life after hallux valgus repair. This study also found that older age correlates with better forefoot narrowing following hallux valgus repair, possibly due to stiffer soft tissues.

## Figures and Tables

**Table 1:** Demographics and preoperative measurements.

Demographics	
Age (years)	63.4 ± 8.3
Gender (N)	
Male	8 (16%)
Female	42 (84%)
Surgery Side (N)	
Left	17 (66%)
Right	33 (34%)
BMI	28.7 ± 4.9

**Table 2:** Changes in preoperative and postoperative hallux valgus angles.

	Preoperative	Postoperative	*p*-Value	Change in Degrees	% of Change
HVA	31.7 ± 6.8	15.6 ± 5.7	<0.001	−16.1 ± 5.4	−50.9 ± 14.0
IMA	13.4 ± 2.8	8.7 ± 2.3	<0.001	−4.7 ± 1.8	−35.1 ± 11.1
BW	9.4 ± 0.6	8.2 ± 0.6	<0.001	−1.2 ± 0.4	−13.0 ± 4.2
STW	10.6 ± 0.7	9.6 ± 0.7	<0.001	−0.95 ± 0.5	−8.9 ± 4.0

**Table 3:** Changes in preoperative and postoperative hallux valgus angles by sex.

	Change in Degrees		*p*-Value	% of Change		*p*-Value
	Males	Females		Males	Females	
HVA	18.0 ± 4.7	15.7 ± 5.6	0.39	52.0 ± 8.8	50.7 ± 14.9	0.94
IMA	4.6 ± 1.8	4.8 ± 1.8	0.82	32.3 ± 10.5	35.6 ± 11.3	0.44
BW	1.4 ± 0.4	1.2 ± 0.4	0.26	13.3 ± 3.7	12.9 ± 4.3	0.65
STW	1.1 ± 0.5	0.9 ± 0.4	0.14	10.2 ± 4.4	8.7 ± 4.0	0.28

**Table 4:** Multivariate analysis of BW change by IMA, HVA, STW, sex, side of surgery, age, and BMI.

	Unstandardized β	Std. Error	Standardized β	t	*p*-Value
IMA	0.28	0.23	0.19	1.24	0.22
HVA	−0.03	0.11	−0.05	−0.28	0.78
STW	0.010	0.11	0.01	0.07	0.95
Sex	0.020	1.81	0.002	0.01	0.99
Side of surgery	1.27	1.24	0.15	1.02	0.31
Age	0.21	0.08	0.42	2.76	**0.01**
BMI	0.21	0.14	0.25	1.52	0.14

**Table 5:** Multivariate analysis of STW change by IMA, HVA, BW, sex, side of surgery, age, and BMI.

	Unstandardized β	Std. Error	Standardized β	t	*p*-Value
IMA	0.18	0.22	0.12	0.82	0.42
HVA	−0.60	0.10	−0.10	−0.59	0.56
BW	0.10	0.12	0.15	0.80	0.43
Sex	−0.14	1.85	−0.01	−0.8	0.94
Side of surgery	−0.22	1.17	−0.03	−0.19	0.85
Age	0.20	0.07	0.42	2.83	**0.01**
BMI	0.19	0.12	0.23	1.55	0.13

## Data Availability

Complete data are available under a confidentiality restriction.

## Abbreviations

HV = hallux valgus, HVA = hallux valgus angle, IMA = intermetatarsal angle, DMMA = distal metatarsal articular angle, BW = bony width, STW = soft tissue width, BMI = body mass index.
